# A Coupled Thermal–Hydrological–Mechanical Damage Model and Its Numerical Simulations of Damage Evolution in APSE

**DOI:** 10.3390/ma9110841

**Published:** 2016-10-31

**Authors:** Chenhui Wei, Wancheng Zhu, Shikuo Chen, Pathegama Gamage Ranjith

**Affiliations:** 1 Centre for Rock Instability and Seismicity Research, School of Resource and Civil Engineering, Northeastern University, Shenyang 110819, China; weichenhui@mail.neu.edu.cn; 2 Faculty of Geosciences and Environmental Engineering, Southwest Jiaotong University, Chengdu 611756, China; shikuochen@foxmail.com; 3 Deep Earth Energy Laboratory, Monash University, Clayton, VIC 3800, Australia; ranjith.pg@monash.edu

**Keywords:** rock damage, thermal–hydrological–mechanical (THM), Äspö pillar stability experiments (APSE), numerical simulation

## Abstract

This paper proposes a coupled thermal–hydrological–mechanical damage (THMD) model for the failure process of rock, in which coupling effects such as thermally induced rock deformation, water flow-induced thermal convection, and rock deformation-induced water flow are considered. The damage is considered to be the key factor that controls the THM coupling process and the heterogeneity of rock is characterized by the Weibull distribution. Next, numerical simulations on excavation-induced damage zones in Äspö pillar stability experiments (APSE) are carried out and the impact of in situ stress conditions on damage zone distribution is analysed. Then, further numerical simulations of damage evolution at the heating stage in APSE are carried out. The impacts of in situ stress state, swelling pressure and water pressure on damage evolution at the heating stage are simulated and analysed, respectively. The simulation results indicate that (1) the v-shaped notch at the sidewall of the pillar is predominantly controlled by the in situ stress trends and magnitude; (2) at the heating stage, the existence of confining pressure can suppress the occurrence of damage, including shear damage and tensile damage; and (3) the presence of water flow and water pressure can promote the occurrence of damage, especially shear damage.

## 1. Introduction

In recent years, the coupled thermal–hydrological–mechanical (THM) behaviour in porous media has been a subject of great interest in many engineering disciplines, such as geothermal energy extraction, recovery of hydrocarbons, safety assessment of waste repositories for radioactive waste, and geological sequestration of CO_2_ [1,2,3,4]. Based on Biot’s theory of the isothermal consolidation of elastic porous media, the phenomenological approach of poroelasticity [5,6], and the mixture theory as described by Morland [7] and Bowen [8], many coupled THM models and computer codes have been developed [3,9,10,11]. In particular, the framework of international projects like DECOVALEX (DEvelopment of COupled models and their VALidation against Experiments) [12,13,14,15], and the tunnel sealing experiment in the Underground Research Laboratory, Canada [16] has dramatically promoted the study of the THM coupling process [17].

In addition, the creation of an excavation-damaged zone (EDZ) is expected around all man-made openings in geologic formations. Macro- and meso-fracturing and, in general, a rearrangement of rock structures, occur in this zone, resulting in drastic changes in the mechanical parameters [13]. For nuclear waste deposits, thermally induced differential stresses near the top of the emplacement drift may cause progressive failure and permeability changes during the first 100 years [14]. During CO_2_ injection, the high injection rate-induced overpressure could also causes a substantial volumetric expansion of the reservoir, resulting in noticeable surface deformation, seismicity, and fault reactivation in the near and far fields [18]. During the extraction of hot dry rock geothermal energy, an artificial storage layer needs to be created by multiple vertical cracks produced by horizontal drilling, which is also related to rock damage evolution under coupled THM conditions [19].

The Äspö pillar stability experiment (APSE) was carried out in the Äspö Hard Rock Laboratory (HRL) to investigate the EDZ development and failure process in country rock mass subjected to coupled excavation-induced and thermal-induced stresses [20]. Mikael et al. [21] used a two-dimensional Boundary Element Method/Displacement Discontinuity Method code to model the pillar response during the planned sequences of excavation–confinement–heating in APSE. Wanne et al. [22] simulated the elastic thermomechanical response for rock mass by using FLAC3D and PFC. As a result of the complexity of the geological conditions at the APSE site, precise predictions of the v-shaped notch location and damage type (shear or tensile damage) are not yet available. In addition, the flow and transport behaviours of water within the developing fractures are dramatically different from those in rocks with existing fractures under the same THM conditions. The permeability of rocks with existing fractures is controlled by fracture aperture variation, while damage evolution in fracturing rocks can induce new channels for fluid flow and cause dramatic changes in permeability [4].

Therefore, an understanding of the formation and subsequent spatial and temporal evolution of EDZs under coupled THM conditions is of great importance when evaluating the long-term performance of underground engineering. In this respect, continuum damage mechanics can provide a theoretical framework for developing damage and fatigue models for rock material. In meso-mechanical models, stresses are assumed to be redistributed due to a decrease in the effective material area [23].

In view of this, the major objective of this paper is to develop a damage-based THM coupling model to study the deformation and failure process of rock under coupled THM conditions. In the mathematical model: (1) statistical distribution will be used to characterize the heterogeneity of rock; (2) the effect of heat convection on temperature variation will be considered; and (3) a gradual damage model rather than an abrupt reduction model will be used, allowing the mesoscopic elements to degrade gradually. In numerical solving of the model, COMSOL Multiphysics (COMSOL AB, Stockholm, Sweden), a powerful partial differential equation-based finite element software, together with programming based on Matlab (MathWorks, Natick, MA, USA), will be carried out to solve the coupled thermal–hydrological–mechanical damage (THMD) model directly. In numerical simulations on APSE, the effect of water pressure distribution on rock damage evolution at the excavation and heating stages will be studied further.

The outline of this paper is as follows. In Section 2, the general conservation equations for the THM coupling model are introduced, followed by the development of the damage evolution equation and its effect on THM parameters. In Section 3, the numerical model development and the determination of the mechanical parameters for the Äspö pillar stability experiment (APSE) are described in detail. In Section 4, the simulation results of damage zone evolution at the excavation stage and the heating stage under different THM conditions are shown. In Section 5, the mechanism of the influence of THM conditions on damage evolution is discussed further.

## 2. Governing Equations

In this section, the governing equations for the coupled thermal–hydrological–mechanical system are derived, based on momentum conservation, mass conservation, and energy conservation, respectively. The rock damage evolution under the coupled conditions and its impact on mechanical parameters are also considered.

Some assumptions are introduced here to simplify the THMD model and to focus on the most relevant processes [24]: (1) small displacements and infinitesimal strains are assumed; (2) the rock material is fully saturated and the fluid (water) does not undergo a phase transformation; (3) thermal equilibrium between the fluid and solid phases is assumed; and (4) the constitutive relationship for the solid skeleton is linearized elastic with mechanical parameter reduction during the damage process.

The mathematical formulation for the fully-coupled THMD system is summarized as follows.

### 2.1. Mechanical Equilibrium Equation

According to thermoporoelastic theory, the constitutive relations for a non-isothermal water-bearing rock material can be expressed in terms of the total stress *σ_ij_* (positive for tension), the total strain *ε_ij_*, water pressure *p* (negative for suction) and temperature change *T* [24] as follows:

(1) $$\sigma_{ij}=2G\varepsilon_{ij}+\frac{2Gv}{1-2v}\varepsilon_{kk}\delta_{ij}-ap\delta_{ij}-K^{\prime }\alpha_{T}T\delta_{ij},$$

where G is the shear modulus and v is Poisson’s ratio. α (≤1) is the Biot’s coefficient, depending on the compressibility of the constituents, and can be defined as $\alpha =1-\frac{K^{\prime }}{K_{s}}$, where $K_{s}$ is the effective bulk modulus of the solid constituent (Pa) and $K^{\prime }=\frac{2G\left(1+v\right)}{3\left(1-2v\right)}$ is the bulk modulus of the porous medium (Pa). $\alpha_{T}$ is the volumetric expansion coefficient of the bulk medium (K^−1^).

Then a modified Navier equation, in terms of displacement $u_{i}$ (m), water pressures p (Pa), and temperature change T (K), can be expressed as

(2) $$Gu_{i,jj}+\frac{G}{1-2v}u_{j,ji}-ap_{,i}-K^{\prime }\alpha_{T}T_{,i}+F_{i}=0,$$

where $\sigma_{ij}$ is the components of the net body force (N·m^−3^) in the *i*-direction $\sigma_{ij}$.

### 2.2. Water Flow Equation

According to Zhou et al. [24], if the water flow follows Darcy’s law:

(3) $$q_{w}=-\frac{k}{\mu_{w}}\left(\nabla p+\rho_{w}g\nabla z\right).$$

The mass conservation equation for the water flow process in porous rock can be expressed as

(4) $$c_{1}\frac{\partial \varepsilon_{v}}{\partial t}-c_{2}\frac{\partial T}{\partial t}+c_{3}\frac{\partial p}{\partial t}=\nabla \cdot \left[\frac{k}{\mu_{w}}\left(\nabla p+\rho_{w}g\nabla z\right)\right],$$

where

(5) $$c_{1}=\alpha =1-\frac{K^{\prime }}{K_{s}},c_{2}=\phi a_{w}+\left(1-\phi \right)a_{s}-\frac{\alpha_{T}K^{\prime }}{K_{s}},c_{3}=\frac{\phi }{K_{w}}+\frac{1-\phi }{K_{s}},$$

where $\varepsilon_{v}$ is the volumetric strain, $\mu_{w}$ is the dynamic viscosity of water (N·s·m^−2^), k is the intrinsic permeability of rock (m^2^), $\rho_{w}$ is the water density (kg·m^−3^), z is the vertical coordinate, g is the gravitational acceleration (m·s^−^^2^), ϕ is the porosity of rock, $a_{w}$ and $a_{s}$ are the thermal expansion coefficient of water and rock grain under constant pore pressure and stress (1/K), respectively, and $K_{w}$ denotes the bulk modulus of water.

### 2.3. Energy Conservation Equation

Based on Fourier’s law, the constitutive relation for heat diffusion can be expressed as

(6) $$q_{T}=-\lambda_{M}\nabla T+\rho_{w}C_{w}\left(T_{ar}+T\right)q_{w}$$

(7) $$\lambda_{M}=\left(1-\phi \right)\lambda_{s}+\phi \lambda_{w},$$

where $q_{T}$ is the thermal flux (J·s^−1^·m^−2^), $C_{w}$ is the water-specific heat constant at a constant volume (J·kg^−1^·K^−1^), and $q_{w}$ is the Darcy velocity as expressed in Equation (3). $\lambda_{M}$, $\lambda_{s}$, and $\lambda_{w}$ are the thermal conductivities of rock medium, rock grain, and water components (J·s^−1^·m^−1^·K^−1^), respectively. The first and second terms on the right-hand side of Equation (6) are the heat conduction by fluid–solid mixture and the heat convection by water flow, respectively.

Assuming thermal equilibrium between the fluid and solid phases, the energy conservation equation can be expressed as [3,6,24,25]:

(8) $$\frac{\partial {\left(\rho C\right)}_{M}\left(T_{ar}+T\right)}{\partial t}+\left(T_{ar}+T\right)K_{w}a_{w}\nabla \cdot q_{w}+\left(T_{ar}+T\right)K^{\prime }a_{T}\frac{\partial \varepsilon_{v}}{\partial t}=-\nabla \cdot q_{T}$$

(9) $${\left(\rho C\right)}_{M}=\phi \left(\rho_{w}C_{w}\right)+\left(1-\phi \right)\left(\rho_{s}C_{s}\right),$$

where ${\left(\rho C\right)}_{M}$ is the specific heat capacity of the water-filled solid medium and $\rho_{s}$ is the mass density of the rock matrix (kg·m^−3^). $C_{w}$ and $C_{s}$ are the water and solid specific heat constants at a constant volume (J·kg^−1^·K^−1^), respectively. $T_{ar}$ is the absolute reference temperature in the stress-free state (K). The first term on the left-hand side of Equation (8) represents the internal heat energy change rate per unit of volume due to temperature change. The second and third terms represent the heat sink due to the thermal dilatation of water and rock, respectively [24].

Assuming constant specific heats ($C_{w}$ and $C_{s}$) and thermal conductivities ($\lambda_{s}$ and $\lambda_{w}$), substituting Equations (6), (7), and (9) into Equation (8) yields

(10) $${\left(\rho C\right)}_{M}\frac{\partial T}{\partial t}-\frac{k}{\mu_{w}}\left(T_{ar}+T\right)\left(K_{w}a_{w}+\rho_{w}C_{w}\right)\nabla^{2}p-\rho_{w}C_{w}\frac{k}{\mu_{w}}\nabla p\cdot \nabla T+\left(T_{ar}+T\right)K^{\prime }\alpha_{T}\frac{\partial \varepsilon_{v}}{\partial t}=\lambda_{M}\nabla^{2}T.$$

### 2.4. Damage Evolution Equation

**Figure 1:**
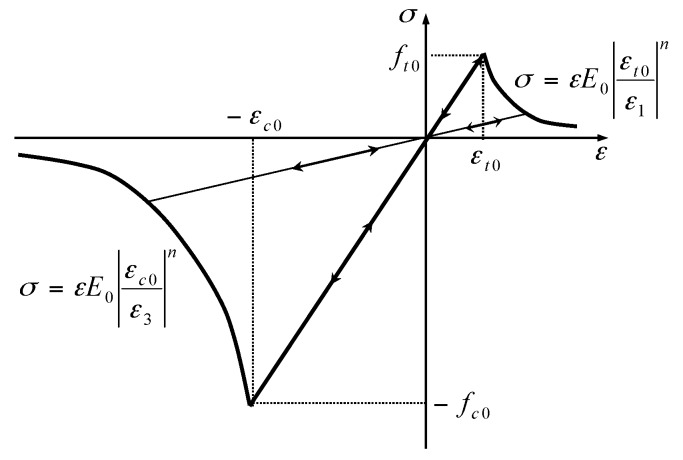
The elastic damage-based constitutive law of elements under uniaxial stress conditions.

As illustrated in Figure 1, when the stress state of rock under coupled THM conditions satisfies the maximum tensile stress criterion or the Mohr–Coulomb criterion, tensile damage or shear damage will occur, respectively. The criteria can be expressed as [26,27,28]:

(11) $$F_{1}\equiv \sigma_{1}-f_{t0}=0\;\text{or}\;F_{2}\equiv -\sigma_{3}+\sigma_{1}\frac{1+\text{sin}\;\varphi }{1-\text{sin}\;\varphi }-fc_{t0}=0,$$

where $\sigma_{1}$ and $\sigma_{3}$ are the first and third principal stresses (Pa), respectively; $f_{t0}$ and $f_{c0}$ are the uniaxial tensile and compressive strength (Pa), respectively; and φ is the internal frictional angle (°).

According to Figure 1, the damage variable D can be calculated as [26,27,28]:

(12) $$D=\{\begin{array}{cccc} 0 & F_{1}<0 & \text{and} & F_{2}<0\\ 1-{\left|\varepsilon_{t0}/\varepsilon_{1}\right|}^{n} & F_{1}=0 & \text{and} & dF_{1}>0\\ 1-{\left|\varepsilon_{c0}/\varepsilon_{3}\right|}^{n} & F_{2}=0 & \text{and} & dF_{2}>0 \end{array},$$

where $\varepsilon_{1}$ and $\varepsilon_{3}$ are the major and minor principal strain; $\varepsilon_{t0}$ and $\varepsilon_{c0}$ are the maximum tensile principal strain and the maximum compressive principal strain when tensile and shear damage occur, respectively; and n is a constitutive coefficient, specified as 2.0. More details can be found in previous publications [26,27,28].

### 2.5. Effect of Damage on THM Parameters

The impact of rock damage on mechanical parameters was also considered, based on the damage theory. The elastic modulus and strength of an element degrade monotonically as damage evolves, and can be expressed as [28,29]:

(13) $$E=\left(1-D\right)E_{0}$$

(14) $$\sigma_{c}=\left(1-D\right)\sigma_{c0},$$

where E and $E_{0}$ are the elastic modulus (Pa) of the damaged and undamaged element, respectively; and $\sigma_{c}$ and $\sigma_{c0}$ are the compressive strength (Pa) of the damaged and undamaged element, respectively. 

The permeability is correlated to the damage variable according to the following exponential function [29]:

(15) $$k=k_{0}\text{exp}\left(\alpha_{k}D\right),$$

where $k_{0}$ is the permeability of the undamaged element and $\alpha_{k}$ is a damage–permeability effect coefficient to indicate the effect of damage on the permeability.

Similarly, the effect of damage on thermal conductivity is given as:

(16) $$\lambda_{s}=\lambda_{s0}\text{exp}\left(\alpha_{\lambda }D\right),$$

where $\lambda_{s0}$ is the thermal conductivity of the undamaged element and $\alpha_{\lambda }$ is a coefficient to reflect the effect of damage on the thermal conductivity.

Equations (2), (4), (10), and (12) represent the fully-coupled thermal–hydrological–mechanical damage response of rock. The coupled non-linear equation is then implemented and solved directly using COMSOL Multiphysics [30], a powerful partial differential equation-based multiphysics modeling environment, together with programming based on Matlab. More details about the implementation and validation of this kind of non-linear equation with COMSOL Multiphysics have been presented in previous publications [25,28].

## 3. Model Setup

### 3.1. APSE Background

As mentioned previously, placing radioactive waste in suitable underground rock formations was one of the early options proposed as a way to remove waste from the biosphere [31]. The Swedish Nuclear Fuel and Waste Management Company (SKB) is undertaking site characterization for spent nuclear fuel. In the KBS-3 concept, the spent fuel is encapsulated in canisters and deposited in vertical 8 m deep, 1.75 m diameter deposition holes excavated in the floor of horizontal deposition tunnels at 400–700 m depths below the surface [32]. At such depths, the excavations induce stress concentrations around the holes and may induce damage in the surrounding rocks. In addition, the decay of used nuclear fuel induces temperature increase, thermal expansion, and thermal stress in surrounding rocks. To address the issue, the Äspö pillar stability experiment (APSE) was carried out in the Äspö Hard Rock Laboratory (HRL) to investigate the EDZ development and failure process in rock mass subjected to coupled excavation-induced and thermally induced stresses [20].

**Figure 2:**
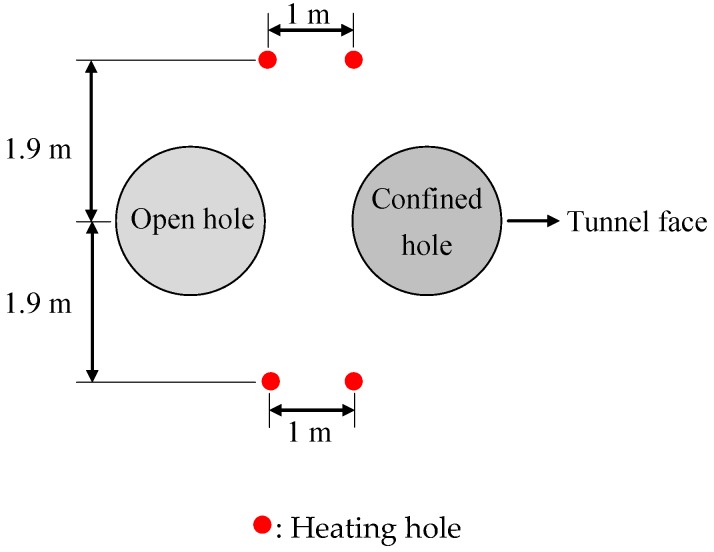
General layout of the two vertical holes and heating holes [20].

A drift with a width of 5 m and height of 7.5 m was excavated first for the experiment. Then, two 1.75 m diameter bore holes were excavated close to each other to create the 1 m wide pillar (Figure 2). A swelling pressure of 0.7 MPa was applied to the confining hole to simulate the backfilling effect of the swelling clay bentonite mixture. Four vertical boreholes were then drilled and four electrical heaters were installed to heat the pillar area (Figure 2).

### 3.2. Determination of Meso-Mechanical Parameters

The mean value of elastic modulus and uniaxial compressive strength (UCS) of intact rock sample in APSE is 76 GPa and 211 MPa, respectively [20]. Because the impact of existing fractures on the pillar damage process is not considered in the numerical model in this paper, reduced mechanical parameters are used instead. According to Barton [33], the reduced elastic modulus and uniaxial compressive strength of rock mass range from 58.5 to 65.6 GPa and 46 to 58 MPa, respectively. Hence, the reduced elastic modulus of 62 GPa and UCS of 52 MPa are used in the present study.

According to the Finite Element Method, the numerical sample will be divided into many elements first to numerically solve the THMD model proposed in Section 2. These elements are called mesoscopic elements here and the mechanical parameters of these elements are called meso-mechanical parameters. The mechanical parameters such as Elastic Modulus and UCS obtained from uniaxial compressive test are called macro-mechanical parameters. In addition, a Weibull distribution, as defined in the following probability density function, is used here to characterize the heterogeneity of rock [29]:

(17) $$f\left(c\right)=\frac{m}{c_{0}}{\left(\frac{c}{c_{0}}\right)}^{m-1}\text{exp}\left[-{\left(\frac{c}{c_{0}}\right)}^{m}\right],$$

where c is the mechanical parameter (such as elastic modulus, UCS, and thermal conductivity) of the mesoscopic elements in the numerical specimen, $c_{0}$ is the scale parameter related to the average of the material parameters, and the shape parameter m reflects the degree of material homogeneity and is defined as a homogeneity index. For higher values of m, the mechanical parameters of more elements are concentrated closer to $c_{0}$ [28].

Hence, a series of uniaxial compressive tests on numerical samples with different meso-mechanical parameters was conducted first. When the macro-mechanical parameters obtained from the numerical sample were close to the reduced parameters from the laboratory experiments, the corresponding meso-mechanical parameters were used in later APSE simulations.

**Figure 3:**
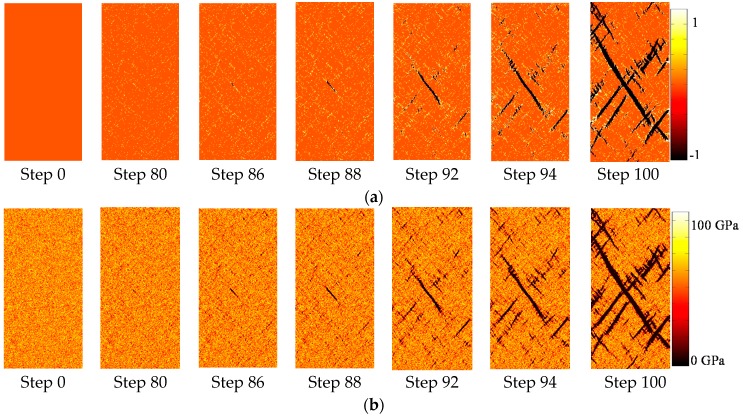
The damage and failure process of the two-dimensional numerical sample during uniaxial compressive test: (**a**) Damage variable evolution; (**b**) elastic modulus evolution.

Figure 3 shows the damage and failure process of a numerical sample (50 mm wide and 100 mm high) under uniaxial displacement of 0.01 mm/step at the upper boundary. Here, a homogeneity index m of 5, a mean elastic modulus of 68 GPa, and a UCS of 119 MPa are used for the mesoscopic elements of the numerical sample. Figure 3a shows the damage variable evolution, in which the tensile damage is represented as negative numbers (−1 ≤ *D* < 0), while the shear damage is represented as positive numbers (0 < *D* ≤ 1), in order to clearly distinguish the two kinds of damage modes. There is no damage in the intact rock sample before compression (Figure 3a, Step 0). As the axial displacement increases, first shear damage initiates, which is randomly distributed in the sample (Figure 3a, Step 80). Then, tensile damage occurs around the shear damage element (Figure 3a, Step 86) and coalescences gradually (Figure 3a, Step 86), leading to the formation of the main macro-fracture (Figure 3a, Step 88). The main macro-fracture located around the middle part of the sample then extends quickly (Figure 3a, Steps 92–94), leading to the final unstable failure of the sample (Figure 3a, Step 100).

According to Figure 1 and Equation (12), each mesoscopic element is damaged gradually under external loading. This means that the damage variable of each element increases monotonically, while damage modes (shear or tensile) can change with stress state. This is why the initial shear damage elements can finally develop into macro tensile fracture.

Figure 3b is the elastic modulus evolution, in which the initial elastic modulus distribution is specified according to a Weibull distribution with a homogeneity index m of 5 (Figure 3b, Step 0). The elastic modulus decreases gradually with damage developing in the mesoscopic elements, shown as black areas in the sample (Figure 3b, Steps 80–100). The reduction of elastic modulus causes stress redistribution around the damaged elements, leading to the extension and coalescence of fractures and the final failure of the sample.

**Figure 4:**
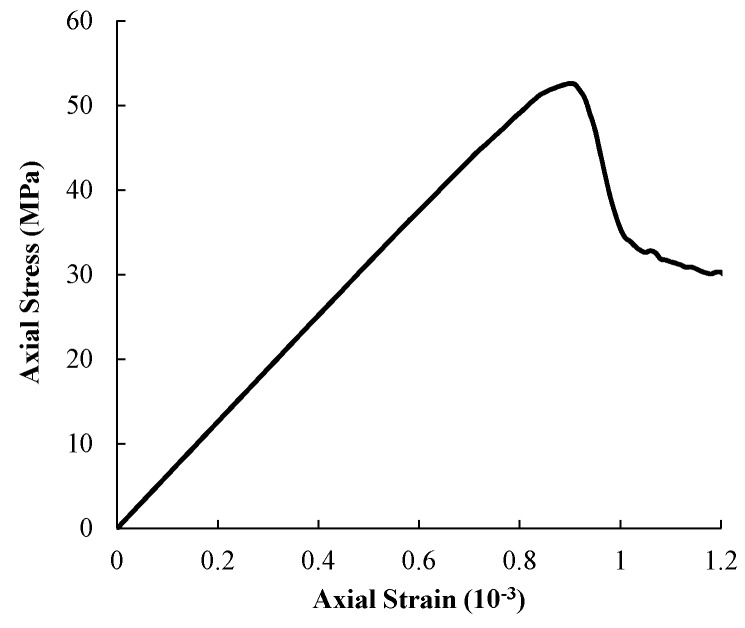
The stress–strain curve of the uniaxial compressive test.

During numerical simulation of uniaxial compressive test, axial strain and stress at the upper boundary at each step are calculated to gain the complete stress–strain curve, from which the macro elastic modulus and UCS can be obtained. Finally, the macro elastic modulus and UCS of 62.8 GPa and 52.6 MPa were obtained (Figure 4) based on the uniaxial compressive test above, which are very close to the reduced parameters of 62 GPa and 52 MPa from the laboratory experiments. Hence, the mean elastic modulus of 68 GPa and a UCS of 119 MPa for the mesoscopic elements are used in later simulations.

Based on Andersson and Martin [20] and the simulations above, the mechanical parameters used in later simulations are listed in Table 1.

### 3.3. Determination of In Situ Stress and Boundary Conditions

Based on hydraulic fracturing, triaxial over-coring, and back analysis, Andersson and Martin [20] give the in situ stress state for the APSE site as shown in Table 2.

**Figure 5:**
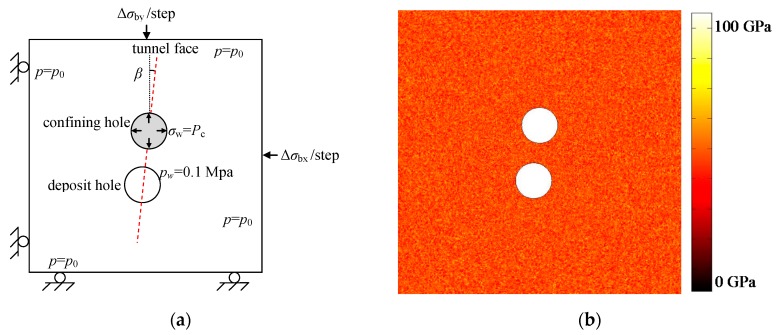
(**a**) The two-dimensional numerical model for APSE at excavation stage; (**b**) initial elastic modulus distribution.

In the horizontal direction, the trends of major principal stress *σ*_1_ and minor principal stress *σ*_3_ in the Äspö 96 coordinate system are 310° and 220°, respectively. The APSE tunnel trend is 46° in the Äspö 96 coordinate system. As a result, the tunnel axis was located approximately 6° off the trend of $\sigma_{3}$, i.e., the tunnel axis was not at right angles to the trend of $\sigma_{1}$ [34]. Hence, a numerical model as shown in Figure 5 is established in this study. One of the benefits of such a model is that only normal stress will exist at the outer boundary of the model and the tangential stress at outer boundary is zero. 

Being limited by the number of mesoscopic elements and computing power for the rock damage process, the actual three-dimensional experiment was simplified to a two-dimensional model. Therefore, pillar stability modeling was performed in one plane of interest, 3.5 m below the tunnel floor. This means that only the major principal stress $\sigma_{1}$ and the minor principal stress $\sigma_{3}$ are considered.

The size of the numerical model is 7.5 m × 7.5 m. The dotted red line in Figure 5a represents the tunnel trend, along which two 1.75 m diameter bore holes are excavated close to each other to create the 1 m wide pillar. The angle between the APSE tunnel trend and the minor principal stress *σ*_3_ is represented by β (equalling 6° for the stress trend in Table 2).

### 3.4. Numerical Model for Excavation Stage

As shown in Figure 5a, at the excavation stage, the vertical boundary stress $\sigma_{by}$ (corresponding to $\sigma_{3}$) and the horizontal stress $\sigma_{bx}$ (corresponding to $\sigma_{1}$) are applied in monotonically increasing mode with an increment $\Delta \sigma_{by}$ of 0.2 MPa and $\Delta \sigma_{bx}$ of 0.6 MPa per step to observe the stable crack propagation process. A confining pressure of 0.7 MPa is applied around the confining hole. An initial water pressure $p_{0}$ of 4.5 MPa is applied at the outer boundary and an atmospheric pressure of 0.1 MPa is applied around the holes. Figure 5b is the elastic modulus distribution with the meso-mechanical parameters listed in Table 1.

**Figure 6:**
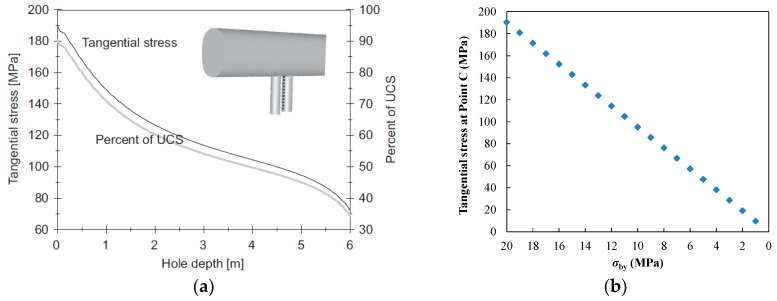
Excavation-induced tangential stress at Point C of the pillar: (**a**) Monitoring result [35], courtesy of SKB (from SKB TR-07-01 (Andersson 2007)); (**b**) simulated result with different boundary stress *σ_by_*.

**Figure 7:**
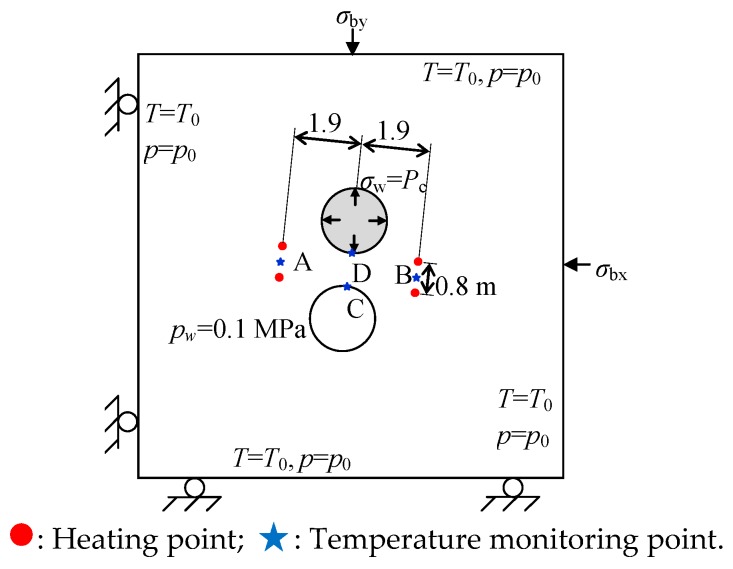
Numerical model for APSE at heating stage.

The excavation of the APSE tunnel causes the in situ stress to redistribute and concentrate within the surrounding rock. The stress state in the pillar decreases with pillar depth. As a result, the precise stress state around the experimental area is difficult to implement. Figure 6a shows the excavation-induced tangential stress measured at different depths [35]. Figure 6b shows our simulation results, from which it can be seen that when $\sigma_{by}$ increases gradually from 1 MPa to 20 MPa, $\sigma_{bx}$ increases gradually from 3 MPa to 60 MPa, and the tangential stress at point C (shown in Figure 7) increases from 10 to 190 MPa. Hence, different $\sigma_{by}$ and $\sigma_{bx}$ can be applied to the outer boundary of the numerical model to simulate the stress states at corresponding depths.

### 3.5. Numerical Model for Heating Stage

After the excavation stage, four heating holes are drilled further to simulate the heating stage (Figure 7). Heater powers used in the experiment and in the numerical simulation are listed in Table 3. The temperature at the outer boundary is set to the initial rock formation temperature $T_{0}$ of 15 °C. A natural air convection boundary is set on the surface of the deposit holes. The heater powers used in the simulation are based on the report and a related study [35,36]. In addition, an initial water pressure $p_{0}$ of 4.5 MPa is applied at the outer boundary and an atmospheric pressure of 0.1 MPa is applied around the holes. Outer boundary stresses $\sigma_{by}$ of 12, 13, and 14 MPa are applied to simulate stress conditions at different depths (corresponding to 3.5, 2.5 and 2.0 m, respectively) in the pillar.

**Figure 8:**
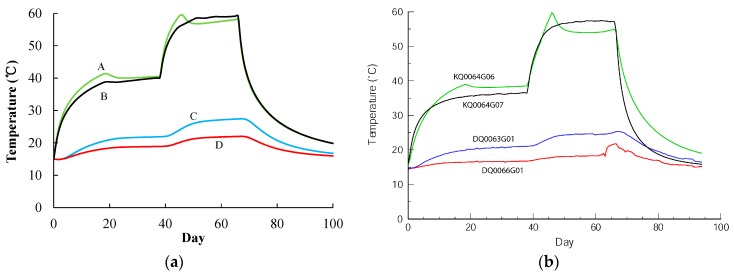
Temperature variation of: (**a**) Simulation result; (**b**) monitoring result [35], courtesy of SKB (from SKB TR-07-01 (Andersson 2007)).

The calculated temperatures at points A, B, C, and D are shown together with the corresponding measurements in Figure 8. The figure shows that a similar temperature distribution is obtained, so that the pillar damage evolution at the heating stage can be simulated further.

It should be noted that a precise description of the temperature field and a detailed discussion on thermal conduction and the thermal convection process based on energy conservation Equation (10) are not provided here, due to the complexity of the APSE site. For example, the monitoring system showed that there was water inflow in three of the cored boreholes and in both of the deposition holes. The water inflow rate in one of the heating holes was up to 5.36 L/min, leading to a dramatic cooling effect in the pillar. Hence, a packer was placed above the fracture and the water was led through the packer pipe out of the hole to weaken the cooling effect of heat convection induced by water flow [35]. All these options make it difficult to precisely recreate the temperature field in simulations. For this reason, obtaining the temperature variation close to the measurement (as shown in Figure 8) and then analysing the pillar damage evolution in such temperature fields are the main purposes of this study.

## 4. Simulation Results

### 4.1. Damage Zone Evolution at Excavation Stage

**Figure 9:**
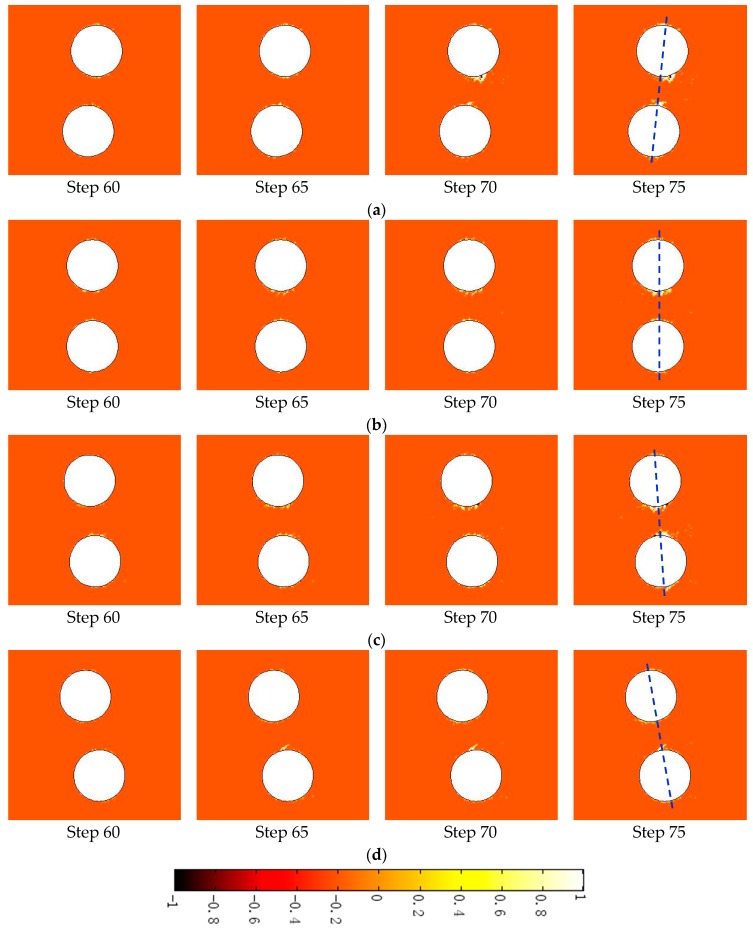
Damage zone evolution under different in situ stress conditions at the excavation stage: (**a**) $\sigma_{1}$ trend of 310°, β = −6°; (**b**) $\sigma_{1}$ trend of 316°, β = 0°; (**c**) $\sigma_{1}$ trend of 320°, β = 4°; (**d**) $\sigma_{1}$ trend of 326°, β = 10°.

Figure 9a shows the damage zone evolution at the excavation stage with β of −6°, in which the tensile damage is represented as negative numbers (−1 ≤ *D* < 0), while the shear damage is represented as positive numbers (0 < *D* ≤ 1). In the numerical model, the “damage zone” represents the mesoscopic element of which the absolute value of damage variable *D* is greater than 1. In physics, the “damage zone” represents the mineral grain or small rock block in which shear damage or tensile damage occurs. At Step 60, shear damage first emerges near points C and D of the shear stress concentration area. With the increase in the external load, this shear damage zone grows gradually (at Step 65) and propagates much deeper into the surrounding rock (at Step 70), resulting in a v-shaped notch in the sidewall (at Step 75). Meanwhile, tensile damage also occurs around the shear damage elements in the v-shaped notch area.

It is noted that the v-shaped notch shows unsymmetrical distribution along the tunnel axis as a result of the deflection angle between the tunnel trend and the in situ major principal stress trend. The shear damage around the open hole is mostly located on the left side of the tunnel axis, while the shear damage around the confining hole is mostly located on the right side of the tunnel axis.

**Figure 10:**
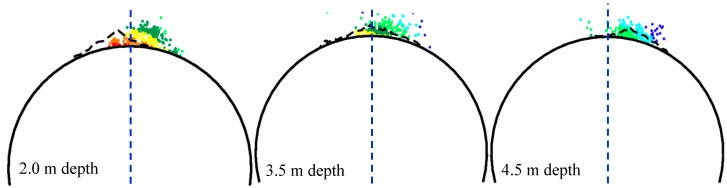
The location of the acoustic emission events at different depths [35], courtesy of SKB (from SKB TR-07-01 (Andersson 2007)).

Figure 10 shows the location of all the acoustic events recorded within a vertical distance of ±0.1 m from each section depth at both the excavation stage and the heating stage [34]. The acoustic events around the open hole are mostly located on the right side of the tunnel axis, which is obviously inconsistent with the simulation results shown in Figure 9a. It can be speculated that the trend of in situ stress (*σ*_1_, *σ*_3_) set in Figure 9a, which is determined according to in situ measurement, is not consistent with the actual in situ stress trend around the deposit holes. The excavation of the drift tunnel has influenced the stress state around the holes, causing not only stress concentration and redistribution but also stress trend deflection.

Hence, further numerical simulations on damage zone evolution under adjusted stress conditions were carried out, in which the trends of in situ stress ($\sigma_{1}$, $\sigma_{3}$) were 316, 226; 320, 230; and 326, 236, respectively (as shown in Figure 9b–d).

For the $\sigma_{1}$ trend of 316°, the shear damage zone around the open hole is distributed uniformly on both sides of the tunnel axis. For the $\sigma_{1}$ trend of 320°, the shear damage around the open hole is mostly located on the right side of the tunnel axis, which is consistent with the location of all the acoustic events. For the $\sigma_{1}$ trend of 326°, the shear damage around the open hole is also located on the right side of the tunnel axis, but the damage area is relatively small and the v-shaped notch is not formed.

A comparison of Figure 9 and Figure 10 indicates that the damage zone distribution with the $\sigma_{1}$ trend of 320° shows relatively good agreement with the location of AE events. This conclusion is also consistent with the research reported by Andersson et al. [35]. Hence, the in situ stress ($\sigma_{1}$, $\sigma_{3}$) trend of 320, 230 is used in the later simulation of damage evolution at the heating stage.

### 4.2. Damage Zone Evolution at Heating Stage

Based on the damage zone distribution at the excavation stage as shown in Figure 9c, the damage zone evolution at the heating stage was simulated. The trends of in situ stress $\sigma_{1}$ and $\sigma_{3}$ were 320° and 230°, respectively.

**Figure 11:**
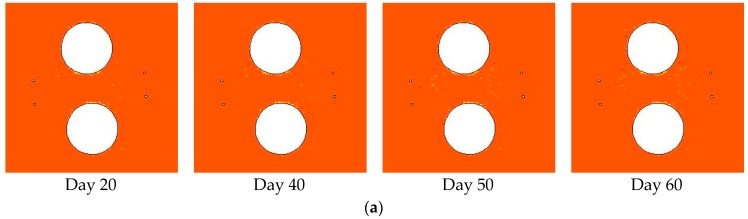

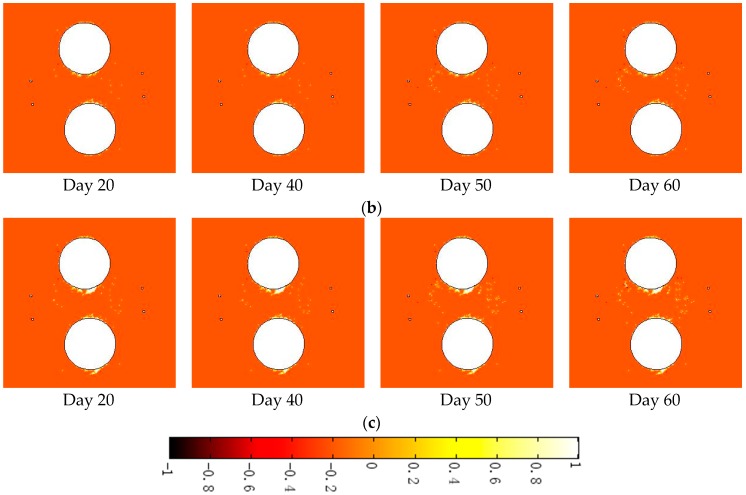
Damage zone evolution under different in situ stress conditions at the heating stage: (**a**) $\sigma_{1}$ = 36 MPa, $\sigma_{3}$ = 12 MPa; (**b**) $\sigma_{1}$ = 39 MPa, $\sigma_{3}$ = 13 MPa; (**c**) $\sigma_{1}$ = 42 MPa, $\sigma_{3}$ = 14 MPa.

In Figure 11a, the initial in situ stress values of $\sigma_{1}$ and $\sigma_{3}$ are 36 MPa and 12 MPa, respectively, corresponding to Step 60 in Figure 9c. Only a few discrete damage points emerge in the area between deposit holes and heating holes, and the shear damage zone near points C and D remain unchanged. In Figure 11b, the initial in situ stress values of $\sigma_{1}$ and $\sigma_{3}$ are 39 MPa and 13 MPa, respectively, corresponding to Step 65 in Figure 9c. More discrete damage points, mainly shear damage, emerge in the area between deposit holes and heating holes, and the shear damage zone near points C and D has barely changed. In Figure 11c, the initial in situ stress values of $\sigma_{1}$ and $\sigma_{3}$ are 42 MPa and 14 MPa, respectively, corresponding to Step 70 in Figure 9c. Much more shear damage occurs in the area between deposit holes and heating holes, and the v-shaped notch also shows a little growth.

**Figure 12:**
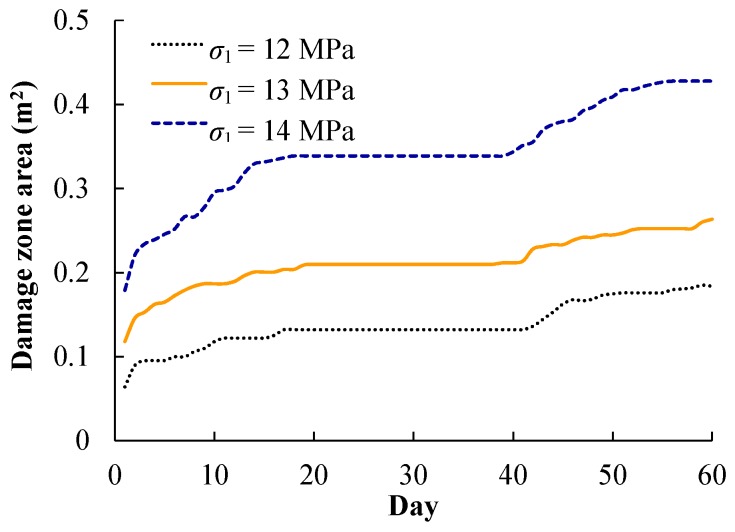
Damage zone area variation over time at the heating stage.

Figure 12 shows the damage zone area variation with time at the heating stage. For initial in situ stresses $\sigma_{1}$ of 36, 39, and 42 MPa, the excavation-induced damage zone areas are 0.06, 0.12, and 0.18 m^2^, respectively. At day 60, the damage zone area increases to 0.18, 0.26, and 0.43 m^2^, respectively. Hence, the heating-induced damage zone areas are 0.12, 0.14, and 0.25 m^2^, which are larger than the excavation-induced damage zone areas, respectively. In addition, the heating-induced damage zone area increases as the initial in situ stress increases, corresponding to a shallower position in the pillar.

**Figure 13:**
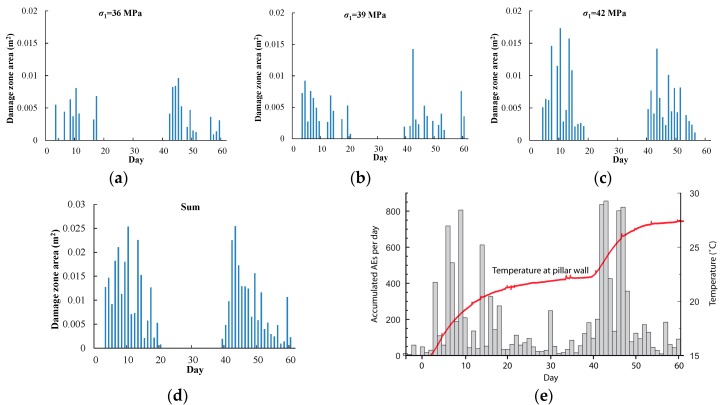
Comparison of damage zone area: (**a**) $\sigma_{1}$ = 36 MPa; (**b**) $\sigma_{1}$ = 39 MPa; (**c**) $\sigma_{1}$ = 42 MPa; (**d**) total damage zone area in simulation; (**e**) accumulated AE events each day [35], courtesy of SKB (from SKB TR-07-01 (Andersson 2007)).

Figure 13 shows the damage zone areas occurring each day under the three different in situ stress conditions (a)–(c), the summation of the daily damage zone area (d), and accumulated acoustic emission (AE) events each day [35]. The figure shows that the damage zone occurs mainly during days 1–20 and 40–60, which is consistent with the increasing temperature. The total damage zone area each day in the simulation is also consistent with the AE events.

### 4.3. Effect of Confining Pressure on Damage Zone Evolution

**Figure 14:**
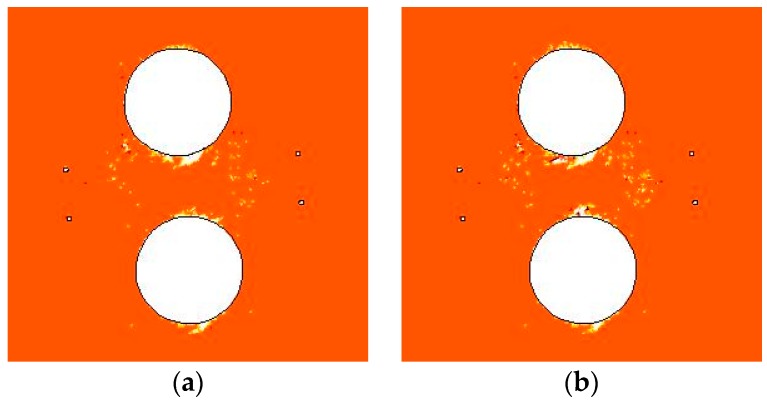
Damage zone distribution at day 60 of heating stage: (**a**) $P_{c}$ = 0.7 MPa; (**b**) $P_{c}$ = 0 MPa.

In underground repositories for radioactive waste, clay bentonite mixtures are usually used to backfill the deposit holes to seal off the canisters. This kind of bentonite mixture swells considerably when exposed to water, resulting in swelling pressure being applied to the rock walls. As shown in Figure 7, a confining pressure $P_{c}$ of 0.7 MPa is applied to the confining hole wall in the above simulations. In the KBS-3 concept the bentonite–soil mixture in the emplacement borehole could generate a swelling pressure of approximately 5 MPa [20]. Hence, numerical simulations on pillar damage evolution at the heating stage with confining pressures of 0, 2, 3, 4, and 5 MPa were carried out to study the impact of confining pressure on damage zone distribution (Figure 14). The initial in situ stress values of $\sigma_{1}$ and $\sigma_{3}$ were 42 MPa and 14 MPa, respectively, corresponding to Step 70 in Figure 9c.

Figure 14 shows the damage zone distribution at day 60 with confining pressure $P_{c}$ of 0.7 and 0 MPa. When confining pressure $P_{c}$ decreases from 0.7 MPa to 0 MPa, more tensile damage occurs at the hole walls, leading to the formation of a deeper v-shaped notch. The shear damage zone in the area between deposit holes and heating holes also increases a little.

**Figure 15:**
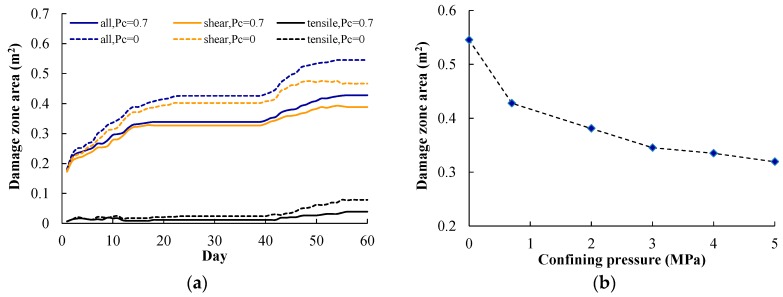
(**a**) Damage variation with $P_{c}$ of 0 and 0.7 MPa; (**b**) damage variation with $P_{c}$ of 0, 0.7, 2, 3, 4 and 5 MPa.

Figure 15a shows the variations of the damage zone area over time, in which the shear damage zone area, the tensile damage zone area, and the total damage zone area are given. For confining pressure $P_{c}$ of 0.7 MPa, the heating-induced total damage zone is 0.43 m^2^, including shear and tensile damage zone areas of 0.39 and 0.04 m^2^, respectively. For a confining pressure $P_{c}$ of 0 MPa, the heating-induced total damage zone is 0.55 m^2^, including shear and tensile damage zone areas of 0.47 and 0.08 m^2^, respectively. When confining pressure increases from 0 to 0.7 MPa, the total, shear, and tensile damage zone areas decrease by about 22%, 17%, and 50%, respectively. This indicates that the shear and tensile damage zones are both suppressed by the confining pressure.

According to Andersson et al. [34], at the heating stage in APSE, the confining pressure began to decrease from day 61 on. During the release of the confining pressure, many AEs were recorded but they died off within two days of the final pressure drop. The numerical simulation results from the FLAC3D model also show that when the confining pressure increases from 0 to 0.8 MPa the crack initiation stress increases by about 4% and the damaged area also becomes smaller [22]. Hence, the conclusion shown in Figure 15 is consistent with the abovementioned measurement [34] and simulations [22].

### 4.4. Effect of Biot’s Coefficient on Damage Zone Evolution

As shown in Equation (4) and Figure 5 and Figure 7, water flow is also considered in the proposed THMD model. The Biot’s coefficient *α* was set to zero in all the above simulations to focus on the effects of excavation, heating, and confining pressure on damage evolution. Hence, a numerical simulation of pillar damage evolution at the heating stage with a Biot’s coefficient α of 0.5 was carried out. The initial in situ stress values of $\sigma_{1}$ and $\sigma_{3}$ were 42 MPa and 14 MPa, respectively, corresponding to Step 70 in Figure 9c.

**Figure 16:**
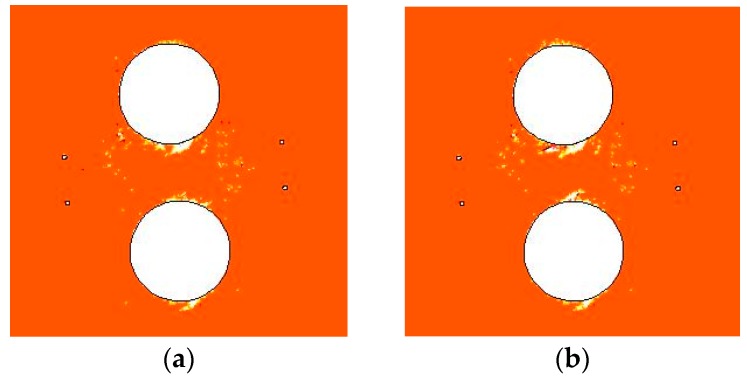
Damage zone distribution at 60 days of heating stage: (**a**) α = 0; (**b**) α = 0.5.

Figure 16 is the damage zone distribution at day 60 with Biot’s coefficients α of 0 and 0.5. When α increases from 0 to 0.5, since the effect of water pressure on pillar deformation is based on the effective stress principle, more tensile and shear damage appears at the hole walls, leading to a deeper v-shaped notch.

**Figure 17:**
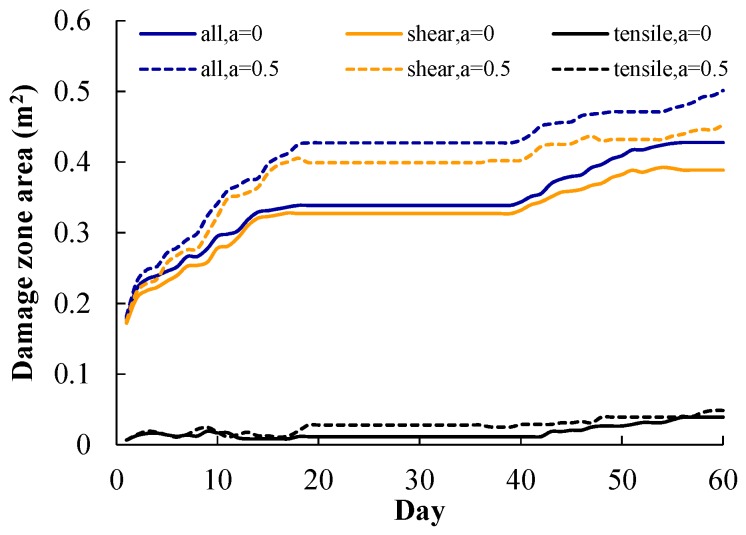
Variation of shear and tensile damage zone area during heating stage.

Figure 17 shows the variations of damage zone area with time, in which the shear damage zone area, tensile damage zone area, and total damage zone area are given. For a Biot’s coefficient α of 0, the heating-induced total damage zone is 0.43 m^2^, including shear and tensile damage zone areas of 0.39 and 0.04 m^2^, respectively. For a Biot’s coefficient α of 0.5, the heating-induced total damage zone is 0.50 m^2^, including shear and tensile damage zone areas of 0.45 and 0.05 m^2^, respectively. When α increases from 0 to 0.5, the total, shear, and tensile damage zone areas increase by about 16%, 15%, and 25%, respectively. This indicates that the shear and tensile damage zones are both promoted by the water pressure.

## 5. Discussion

In this paper, a damage-based model for rock under coupled thermal–hydrological–mechanical conditions is proposed, in which the heterogeneity of rock is also characterized using Weibull distribution. The proposed model is then implemented in, and solved by, COMSOL Multiphysics, a powerful PDE-based multiphysics modeling environment.

The simulation results for the damage zone distribution at the excavation stage (Figure 9) and the heating stage (Figure 11) show that the location and depth of the v-shaped notch are mainly controlled by the trend and magnitude of in situ stress ($\sigma_{1}$, $\sigma_{3}$) respectively, while heating-induced stress is mainly responsible for the damage zone located in the area between the deposit holes and heating holes. The damage zone distribution with in situ stress ($\sigma_{1}$, $\sigma_{3}$) trends of 320, 230 shows relatively good agreement with the AE location. In addition, the v-shaped notch under in situ stress ($\sigma_{1}$, $\sigma_{3}$) of (36 MPa, 12 MPa), corresponding to the 3.5 m depth section in Figure 10, is still not formed at the end of the heating stage (Figure 11a, Step 60). The main reasons for the above discrepancies between our simulations and monitoring are likely: (1) simplification from a three-dimensional jointed rock mass to a two-dimensional heterogeneous rock; (2) simplifications of the horizontal in situ stress directions; (3) absence of the middle principal stress in the damage criterion; and (4) simplification of thermal and hydraulic boundary conditions.

Although the damage zone area that developed each day shows good agreement with the AE events, as shown in Figure 13, especially during days 1–20 and 40–60, the simulation result during days 21–39 is relatively different from the monitoring. After the temperature increasing stage from day 1 to 19, the temperature around the deposit holes reaches about 22 °C and basically remains stable during days 21–39. A relatively small number of AE events are recorded during this time. However, in our simulation no damage occurs at the same time. It is likely that the actual damage process of rock, especially of jointed and heterogeneous rock masses, takes a relatively long time to complete, including damage in local mesoscopic minerals, stress redistribution, permeability variation, water pressure, and rock temperature variation. This time-dependent effect of the rock mass, however, is not considered in our simulations.

Figure 15b shows that when confining pressure $P_{c}$ increases from 0 to 0.7, 2, 3, 4, and 5 MPa, the total damage zone area decreases from 5.5 to 4.3, 3.8, 3.5, 3.4, and 3.2 m^2^, decreasing by about 22%, 30%, 37%, 39%, and 41%, respectively. This indicates that the heating-induced damage zone area decreases as the confining pressure increases, but the decreasing amplitude declines gradually as the confining pressure increases from 0 to 5 MPa. Considering the fact that the confining pressure applied on the deposit hole wall can counteract temperature increase-induced swelling, the finding in Figure 15b is understandable, because confining pressure’s inhibition on damage development is related to the original increment of temperature and thermal stress.

Figure 17 indicates that shear and tensile damage are both promoted by water pressure. The increments of total, shear, and tensile damage are 0.07, 0.06, and 0.01 m^2^, respectively. There is a relatively larger increment of shear damage because the effective stress has the effect of translating the Mohr circle to the left by the amount αp. The increment of tensile damage, however, is relatively small as a result of the compression induced by the in situ stress. Hence, the deformation and reopening of pre-existing fractures and the impact on water pressure distribution will have a great influence on damage evolution, which will be the subject of future research.

Notwithstanding the model limitations mentioned above, the results of the presented modeling work indicate that the application of numerical simulation tools provides a valuable insight regarding the evaluation and prediction of underground nuclear waste repositories under coupled THM conditions. To overcome the given limitations step-by-step is an essential part of future work [17].

## Figures and Tables

**Table 1:** Parameters used in the numerical simulation [20,33].

Mechanical Property	Value
Reduced elasticity modulus	62 GPa
Poisson’s ratio	0.25
Reduced UCS	52 MPa
Homogeneity index	5
Mean elastic modulus of mesoscopic element	68 GPa
Mean UCS of mesoscopic element	119 MPa
Mean tensile strength of mesoscopic element	12 MPa
Frictional angle	49°
Density of rock	2750 kg·m^−3^
Volume heat capacity	770 J·kg^−1^·K^−^^1^
Thermal conductivity	2.6 W·m^−^^1^·K^−^^1^
Linear expansion	7 × 10^−6^ K^−^^1^
Initial temperature	15 °C
Permeability	1 × 10^−^^17^ m^2^
Dynamic viscosity of water	1 × 10^−^^3^ Pa·s

**Table 2:** Back-calculated stress state for the APSE site [20].

In Situ Stress	$\sigma_{1}$	$\sigma_{2}$	$\sigma_{3}$
Magnitude (MPa)	30	15	10
Trend (Äspö 96)	310	90	220
Plunge (degrees from horizontal)	0	90	0

**Table 3:** Heater powers used in the experiment and in the model [35,36].

Days from Heater Startup	Output (W/m)			
	Left Side Heaters		Right Side Heaters	
	Experiment	Model	Experiment	Model
0	200	200	200	180
18	170	160	200	160
38	354	348	400	320
42	354	348	400	320
46	263	263	400	300
52	263	263	400	280
59	263	263	400	270
66	0	0	0	0
